# Applicability of near-infrared reflectance spectroscopy (NIRS) for determination of crude protein content in cowpea (*Vigna unguiculata*) leaves

**DOI:** 10.1002/fsn3.7

**Published:** 2013-01-08

**Authors:** Erick K Towett, Merle Alex, Keith D Shepherd, Severin Polreich, Ermias Aynekulu, Brigitte L Maass

**Affiliations:** 1 Department of Crop Sciences: Agronomy in the Tropics, Georg-August University of GoettingenGoettingen, Germany; 2 World Agroforestry Centre (ICRAF)Nairobi, Kenya; 3 Institute for Crop and Soil Science, JKI – Federal Research Centre for Cultivated PlantsBraunschweig, Germany; 4 International Potato Center (CIP)Apartado, Lima, Peru; 5 International Center for Tropical Agriculture (CIAT)Nairobi, Kenya

**Keywords:** African vegetable, calibration, near-infrared reflectance spectroscopy, nutritional quality, prediction

## Abstract

There is uncertainty on how generally applicable near-infrared reflectance spectroscopy (NIRS) calibrations are across genotypes and environments, and this study tests how well a single calibration performs across a wide range of conditions. We also address the optimization of NIRS to perform the analysis of crude protein (CP) content in a variety of cowpea accessions (*n* = 561) representing genotypic variation as well as grown in a wide range of environmental conditions in Tanzania and Uganda. The samples were submitted to NIRS analysis and a predictive calibration model developed. A modified partial least-squares regression with cross-validation was used to evaluate the models and identify possible spectral outliers. Calibration statistics for CP suggests that NIRS can predict this parameter in a wide range of cowpea leaves from different agro-ecological zones of eastern Africa with high accuracy (*R*^2^cal = 0.93; standard error of cross-validation = 0.74). NIRS analysis improved when a calibration set was developed from samples selected to represent the range of spectral variability. We conclude from the present results that this technique is a good alternative to chemical analysis for the determination of CP contents in leaf samples from cowpea in the African context, as one of the main advantages of NIRS is the large number of compounds that can be measured at once in the same sample, thus substantially reducing the cost per analysis. The current model is applicable in predicting the CP content of young cowpea leaves for human nutrition from different agro-ecological zones and genetic materials, as cowpea leaves are one of the popular vegetables in the region.

## Introduction

Near-infrared reflectance spectroscopy (NIRS) has been used to measure composition because of the combination and overtones in the NIR region from the fundamental vibrations in mid-infrared reflectance region, and the simultaneous prediction of properties comes from the chemometrics applied to the spectral and chemical matrices (Barton et al. 1992; Foley et al. 1998; Kawano 2002). The technique offers several advantages such as rapid determination, little or no preparation of samples, nonconsumptive analysis, no consumption of reagents, and low costs of analyses compared with the conventional methods (Bruno-Soares et al. 1998; Foley et al. 1998; Kawano 2002; Shepherd and Walsh 2007; Pojić et al. 2010; Decruyenaere et al. 2012). NIRS offers good predictive power, but care must be taken in the calibration stage to guard against prediction failure. Thus, the applicability of the NIRS technique is limited by the need to have an experienced and knowledgeable NIR scientist to develop and update the prediction models, check for seasonal bias and correct for it, and generally maintain and update the models. In theory, selection of samples that cover the range of spectral variation in a data set should be sufficient; however, the calibration should include not only possible genotypic variation but also all sources of variance (Foley et al. 1998). Researchers have aimed at developing universal calibrations embracing a whole commodity group, even like all forages, (Undersander et al. 2005; Decruyenaere et al. 2009) instead of favoring individual calibrations for different species, locations, years, or seasons (Míka et al. 2003; García and Cozzolino 2006). Although scientists have investigated reflectance spectroscopy for several decades, the technology has not been widely taken up and routinely applied in nutritional quality studies in the African context.

Near-infrared reflectance spectroscopy has been successfully applied to predict the nutritive value of forages with *R*^2^ values of 0.90 or higher, and standard errors well within laboratory errors have been reported for crude protein (CP, in%) in a variety of forages, feed stuffs, and young legumes and grasses (Lenné et al. 2003; Stuth et al. 2003; Cozzolino and Morón 2004; García and Cozzolino 2006; Tefera 2006). Some studies also included cowpea (*Vigna unguiculata* (L.) Walp.) as a forage legume (Brink and Fairbrother 1988; Lenné et al. 2003). In Eastern and Southern Africa, however, tender cowpea leaves are regularly picked and eaten as a vegetable, like spinach and, as such, they can significantly contribute to nutrition quality (Nielsen et al. 1997; Keller et al. 2006; Tefera 2006). Data on nutritional quality of African leafy vegetables, such as cowpea leaves, is insufficient and often in need of validation because not many analyses are available for a number of reasons, including the lack of functional laboratories and lack of resources for field sampling campaigns. The use of NIRS as a possible technique to circumvent some of these problems needs to be explored.

A NIRS calibration for CP in young legume leaves has previously been developed within the framework of the Promotion of Neglected Indigenous Leafy and Legume Vegetable Crops for Nutritional Health in Eastern and Southern Africa (ProNIVA) project (Tefera 2006). The available calibration model from the previous study (named initial model here) was based on 103 leaf samples selected from a broad spectrum of cowpea and lablab (*Lablab purpureus* (L.) Sweet) accessions (*n* = 939) grown under three different environments in Tanzania (Tefera 2006). The initial calibration model was applied to 189 samples of cowpea and lablab accessions grown under different field conditions in Malawi as well as under greenhouse and outdoor conditions at the Department of Crop Sciences' Institute of Agronomy in the Tropics (DNPW-IAT) of University of Göttingen, Germany (Magesa 2006; Malidadi 2006). The obtained coefficient of determination for predicted versus reference CP in the samples grown under greenhouse and outdoor conditions in Germany was relatively good (*R*^2^ = 0.84; Magesa 2006). Similarly, an *R*^2^ value of 0.94 for CP (%) of 20 cowpea leaf samples originating from an experiment in Malawi was obtained (Malidadi 2006). Despite acceptable *R*^2^ values for CP in cowpea leaves when using the initial calibration model to predict new samples, the global-H values and biases were not acceptable (>3). There was clearly a need to further develop the robustness of the CP calibration model for general use in young vegetable cowpea leaves. The objective of this study was to examine the applicability of NIR to predict CP content in young cowpea leaves and to improve upon the initial calibration model with more data. There is also uncertainty on how generally applicable calibrations are across genotypes, growth environments, and this study tests how well a single calibration performs across a wide range of conditions.

## Materials and Methods

### Sample collection

Samples collected by previous M.Sc. and Ph.D. studies of the ProNIVA (phase II) project in five environments in Tanzania namely Arusha, Mwanga, Morogoro, Dodoma, and Majimoto and one environment in Serere, Uganda, were obtained for this study. In addition, samples of cowpea leaf samples collected from formal and informal markets in Nairobi, Kenya, and Arusha, Morogoro, and Dodoma in Tanzania, as well as from farmers' fields in Arusha, Morogoro, and Dodoma in Tanzania (Towett 2008). Different agronomic evaluations of the cowpea as a leafy vegetable were conducted in the different environments, and the experimental trials were all established in a randomized complete block design with three replicates in Morogoro and Mwanga and four replications in all the other trial stations. One trial was established on-station and the same repeated on-farm except for the Morogoro and Serere trials, which were established on-station (Towett 2008; Polreich 2010). Spacing between plots was 1 m and 1.5 m between repetitions, while spacing for cowpea in each trial plots was 75 cm by 15 cm. The duration of each trial was approximately 4 months depending on the environmental conditions. Details of all the agronomic practices are mentioned elsewhere (Kabululu 2008; Towett 2008; Okonya 2009; Polreich 2010).

## Sample processing

For the present study, a set of 561 cowpea leaf samples representing a wide range of environments in Tanzania and Uganda, in which cowpea is typically grown, as well as genotypic variation (38 accessions, including released cultivars, landraces, experimental lines, and research materials) were collected in 2008 and sun-dried immediately after harvest for 3 days in special bags made out mosquito nets. Dried samples were milled using a standard laboratory grinder (Mikro-Schlagmühle Culatti: Janke and Kunkel KG, JKA-Werk, Staufen, Germany) to pass through a 1-mm sieve.

## Spectra acquisition and reference analysis

The milled, sun-dried samples were scanned, about 3 months after harvesting, using a Foss 6500 NIR Composite Monochromator (Foss NIRS Systems, Silver Spring, MD) at University of Göttingen, Germany. Small ring sample cups with a quartz window installed, designed for the analysis of dry, ground products, were employed in conjunction with the Foss NIR spectrometer. For reference analysis of nitrogen (N), an Elemental Analyzer (Vario EL III Elementar Analysensysteme GmbH, Hanau, Germany) was employed. About 3–5 mg of each cowpea leaf sample was weighed into tin capsules measuring 4 × 6 mm, using a Sartorius Micro scale (Sartorius AG, Göttingen, Germany) at the laboratory of the Institute of Agronomy in the Tropics, Crop Sciences Department of University of Göttingen, Germany. One to two milligrams of acetanilide (Merck KGaA, Darmstadt, Germany) was also weighed into tin capsules as a test standard for elementary analysis. The measured N content was multiplied by a factor of 6.25 to estimate CP content in cowpea leaves (AACC International 1999).

## Data analysis

Spectral analyses and development of calibration models were conducted at Julius Kühn Institute (JKI), Braunschweig, Germany. The spectral data were subjected to a modified partial least-squares regression (MPLR) with internal cross-validation and scatter correction using standard normal variate transformation and detrending using the WinISI II version 1.50 software (Infrasoft International, Port Matilda, PA). The WinISI software compares the samples on the basis of the Mahalanobis distance (H) values, that is, the distances in spectral data from the mean of the population and from each other, and selects that offer the most diverse groupings of spectra. The WinISI principle is that, if two or more samples are very similar spectrally, only one of them is needed. Principal component analysis (PCA) was performed on the spectral data to get an overview of the spectral variability in the sample set and as a basis for outlier detection. The spectral range used was 1100–2498 nm and the mathematical treatment applied was (1,4,4,1), where the first digit indicates the number of the derivative (1 is first derivative of log (1/*R*)); the second is the gap in data points over which the derivative is calculated; the third indicates the number of data points used in the first smoothing; and the fourth is the second smoothing (Stuth et al. 2003; González-Martín et al. 2006). The effect of the first derivative on the spectra is to show only component absorption (Foley et al. 1998; Míka et al. 2003). Several three-dimensional plots were produced with WinISI software to observe whether any trends were apparent in the spectral characteristics of each sample set used.

## Calibration development

One hundred and sixty-seven samples were selected using MPLS based on their spectral variation (a 26 PCA-Factor-model with a cutoff value of 0.6 was used) to reflect all the 561 spectra. Spectral data from the selected 167 samples were then taken to expand the spectral variation of the initial calibration model based on 103 samples (Tefera 2006) to give a new calibration model (*n* = 261). The development of the initial calibration model was conducted under the same conditions for reference analysis and NIRS. There is debate whether a calibration procedure should be done by separating a pool of samples into independent calibration data sets and validation data sets or by cross-validation. Several studies showed that the calibration/validation procedure and cross-validation procedure are equally valid (Shenk and Westerhaus 1993a,1993b; Coûteaux et al. 2003; Moron and Cozzolino 2004; Ludwig et al. 2008). In our study, we decided to use cross-validation because the initial calibration model available was developed using the cross-validation procedure, and we decided to use the same approach in order to expand its variation. Another reason for using cross-validation was the number of samples selected for our study (*n* = 167), which is sufficient for a cross-validation, but too small to be split into separate calibration and validation sets. The rational for using this sequential approach was to develop a robust calibration model and apply it on various sample sets to see the effect of adding samples from different environments on the prediction of the same samples and independent ones that were not contained in the new calibration model. The new calibration model obtained was also validated against independent samples sets originating from field conditions in Malawi (Malidadi 2006), Uganda (Okonya 2009), and Tanzania (Kabululu 2008) and greenhouse conditions in Germany (Magesa 2006), to check its prediction accuracy. Comparing the laboratory reference values with those predicted by NIRS with cross-validation revealed the goodness of the predictions obtained for CP. The ratio performance deviation (RPD) calculated as the standard deviation/standard error of prediction (SD/SEP) was also used to evaluate the calibrations (RPD > 3 are good for screening purposes and RPD > 5 good for analytical applications (Williams 2007)). Locher et al. (2005) cautioned that RPD interpretations are strongly dependent on the number and distributions of the reference values and, therefore, cannot be regarded ultimate criterion for prediction quality. Finally, the goodness of calibration models was, therefore, assessed by the coefficient of determination in calibration (*R*^2^) and the standard error of cross-validation (SECV) with the optimum calibration model being chosen on the basis of the minimum SECV and the highest *R*^2^.

## Results and Discussion

### Results of reference analysis

Crude protein content of all samples collected in Tanzania and Uganda was assessed by reference analysis, and the samples were divided into batches so as to check for differences in CP (%) content caused by different environments, or leaf harvests (Table 1). Samples from Majimoto, Tanzania, had the lowest mean CP content while samples from Arusha, Tanzania, had the highest (Table 1). However, mean CP content in the second leaf harvest was found to be the lowest, whereas the fifth leaf harvest had the highest mean CP content (Table 1).

**Table 1:** Results of the reference analysis of leaf crude protein (%) content across different cowpea accessions collected from various environments in Tanzania and Uganda, as well as harvests and processing and description of laboratory replicate variances.

	*N*	Mean	SD	Min	Max
Overall	561	33.4	3.4	21.5	43.7
Location					
AVRDC-RCA	173	34.8	4.0	25.5	43.7
Dodoma	39	34.3	1.4	31.5	36.9
Majimoto	39	30.9	2.2	26.7	36.6
Mwanga	66	34.0	2.0	28.7	39.2
Serere	31	33.0	2.1	29.4	38.6
Morogoro	36	34.4	2.4	30.0	38.8
Grinder					
Coffee	130	33.2	4.0	21.8	43.7
Laboratory	431	33.5	3.3	21.5	42.6
Leaf harvest^1^					
First	87	37.1	2.7	31.4	43.7
Second	285	32.0	2.5	26.2	38.8
Third	41	34.1	3.2	25.5	39.3
Fourth	36	34.9	2.7	27.3	39.2
Fifth	4	37.4	1.0	36.1	38.5

1 The total number of “leaf harvest” is 453 instead of 561 because the cowpea leaf samples obtained from markets could not be classified according to this category as most of samples obtained included uprooted whole plants or cowpea leaves that could not be determined whether they were from first, second, etc., harvest.

## Spectral analyses

**Figure 1:**
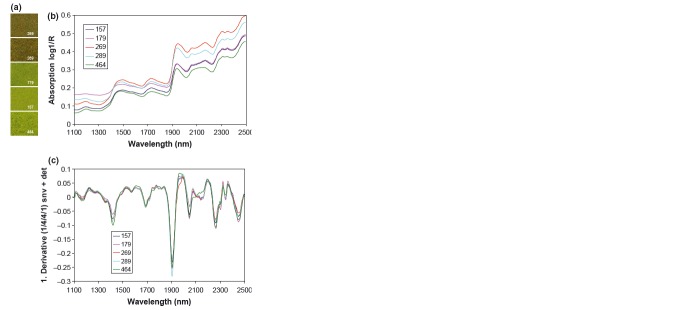
Examples of the variation in five cowpea leaf samples scanned by near-infrared reflectance spectroscopy; (a) visual presentation; (b) spectral absorption; and (c) first derivative (D1) of spectral absorption calculated using mathematical treatment (1,4,4,1; first derivative, gap over which derivative was calculated, number of data points used in first smoothing and in second smoothing).

The shape of the spectra and the rate of change in slope with wavelength convey chemical information contained in the spectra. Examples of typical NIR spectra obtained from five different cowpea samples are shown in Figure 1a and b. Therefore, the first derivative (Fig. 1c) of log 1/*Reflectance* is useful in resolving overlapping bands as well as minimizing the effect of particle size (Foley et al. 1998; Míka et al. 2003).

## Development of crude protein calibration

**Figure 2:**
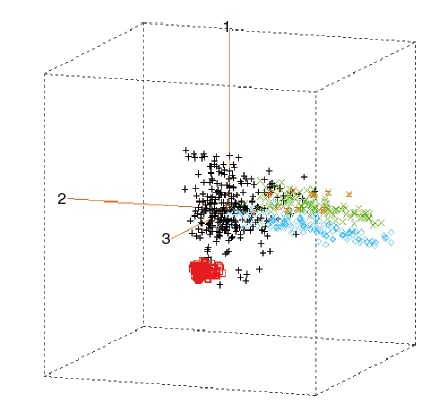
Comparison of different sample sets with the subset of samples used to develop the new calibration model by their first three principal components based on spectral variability of ground cowpea and lablab leaves collected from Tanzania, Kenya, Uganda, and Malawi, as well as under greenhouse and outdoor conditions in Göttingen, Germany. Black (+), Initial + Current (*n* = 274; used to develop new model); red (□), Malawi (*n* = 126); light blue (◊), Germany freeze-dried (*n* = 145); green (x), Germany oven-dried (*n* = 117); orange (z), Germany sun-dried (*n* = 14).

Figure 2 shows a 3D display of the first three PC scores derived from the spectra of the different sample sets available for this study, including those from the previous study used to develop the initial calibration model (Tefera 2006), as well as the samples available from two previous studies (Magesa 2006; Malidadi 2006), used for independent validation. Apparently, large spectral variability was added to that available when the initial calibration model (Tefera 2006) was developed, due to different growing seasons, further genetic materials, and environmental conditions. Individual sample sets used for validation purposes can also be distinguished; for example, the Malawi sample set (Malidadi 2006) overlaps with the main population used in this study, while samples obtained from Germany (Magesa 2006) expand variability into a different direction in the 3D display of the PC scores.

## Model evaluation

Calibration models were evaluated by cross-validation, as well as independent validation sets, and the predicted values gave validation errors that were reflected in the SECV values (Table 2).

**Table 2:** Modified partial least-squares statistics of calibration and cross-validation for two calibration models developed using near-infrared reflectance spectroscopy (NIRS) for crude protein content (CP, in%) based on different sample sets and combinations of cowpea leaves from Eastern Africa

Model	*N*	Mean	SD	Min	Max	SECV	*R*^2^	G-H	RPD
Initial^1^	103	30.9	5.45	14.7	47.3	0.60	0.82	3.46	11.2
New	261	32.1	4.52	18.6	45.7	0.74	0.93	0.71	6.84

*N*, number of spectra in the calibration set; Mean, estimated by NIRS (expressed as CP, in %); SD, standard deviation; Min, lowest value of reliably estimating samples; Max, maximum value of reliably estimating samples; RPD, ratio performance deviation; SECV, standard error of cross-validation; *R*^2^, determination coefficient of calibration; G-H, global-H value, where H is the Mahalanobis distance.

1 Initial calibration model (Tefera 2006) of 107 samples had four outliers removed.

Predicted CP contents of all samples collected in Kenya, Tanzania, and Uganda ranged from 18.6% to 45.7% (Table 2). There was also considerable variation within the same accession across environments and/or harvests (data not shown). Values for cowpea leaf CP were within the ranges (25–35%) reported by other researchers (Kochhar et al. 1988; Carnovale et al. 1991; Nielsen et al. 1997; Ahenkora et al. 1998; González-Martín et al. 2006; Magesa 2006; Malidadi 2006; Tefera 2006; Kabululu 2008). However, the results of prediction of samples used in this study (Table 2) suggest that leaves of some varieties may even have higher or lower CP contents than those that have been previously reported. The broad range observed appears not only due to genetic but also due to environmental variation. Overall, these results support the suggested high nutritional value of cowpea leaves as a vegetable (Nielsen et al. 1997).

**Table 3:** Determination coefficient of calibration (*R*^2^) and mean global-H values for two different near-infrared reflectance spectroscopy calibration models developed for crude protein content (CP, in%) using different sets of cowpea leaf samples available for this study

		Initial model		New model	
Sample sets	*N*	*R*^2^	Global-H	*R*^2^	Global-H
Overall	561	0.82	3.46	0.93	0.71
Location					
Arusha, Tanzania	173	0.92	2.58	0.95	0.58
Dodoma, Tanzania	39	0.53	6.42	0.53	0.87
Majimoto, Tanzania	39	0.70	3.22	0.82	0.67
Morogoro, Tanzania	36	0.78	3.66	0.84	0.62
Mwanga, Tanzania	66	0.86	2.82	0.91	0.71
Serere, Uganda	31	0.78	2.97	0.85	0.51
Markets/farmers					
Markets, Kenya and Tanzania	93	0.94	4.36	0.95	1.02
Farmers, Tanzania	84	0.70	3.52	0.70	0.76

*N*, number of samples (included 167 samples from the initial calibration model); *R*^2^, determination coefficient of calibration; G-H, global-H value, where H is the Mahalanobis distance.

## Application of the crude protein models

When comparing the two calibration models, the major improvement of *R*^2^ value was in the new model compared with the initial one. This was because the samples used to develop the new model were selected based on spectral variation. Similarly, the average global-H value reduced substantially (Table 3), confirming the increased robustness of the new model. In the new calibration model, the overall global-H value was 0.7 and in the different sets, they were not higher than 1.0 (Table 3).

**Table 4:** Prediction statistics when applying two near-infrared reflectance spectroscopy calibration models developed for crude protein content (CP, in%) in young cowpea leaves to different sets of cowpea and lablab (Magesa 2006; grown both under greenhouse conditions and outdoors in Göttingen, Germany), leaf samples independent from the calibration sets

		Initial model		New model	
Sample sets	*N*	*R* ^2^	Global-H	*R* ^2^	Global-H
Africa					
Malawi: field trial (Malidadi 2006)	126	n.a.	8.69	n.a.	4.63
Selected samples for reference analysis	20	0.94	8.21	0.95	4.52
Tanzania: Dodoma (Kabululu 2008)	473	n.a.	8.05	n.a.	4.29
On-farm, used for reference analysis	79	0.19	10.54	0.13	5.73
On-station, used for reference analysis	41	0.43	7.69	0.57	4.08
Samples from five different leaf harvests	38	n.a.	n.a.	0.85	1.56
Uganda: Serere (Okonya 2009)	42	n.a.	n.a.	0.88	0.69
Samples selected for spectral variability	20	n.a.	n.a.	0.87	0.73
Selected for experimental settings' diversity	22	n.a.	n.a.	0.88	0.07
Germany: Goettingen (Magesa 2006)					
Freeze-dried^1^	61	0.77	8.25	0.74	3.35
Oven-dried^1^	117	0.29	5.97	0.33	3.25
Sun-dried^1^	14	0.98	4.48	0.97	1.95

*N*, number of samples; *R*^2^, determination coefficient of calibration; Global-H, global-H value, where H is the Mahalanobis distance; n.a., not available (the calibration models were not applied to the respective sets with no data available).

1 Freeze-, oven-, and sun-dried samples included 61, 48, and 7 lablab leaf samples, respectively.

**Figure 3:**
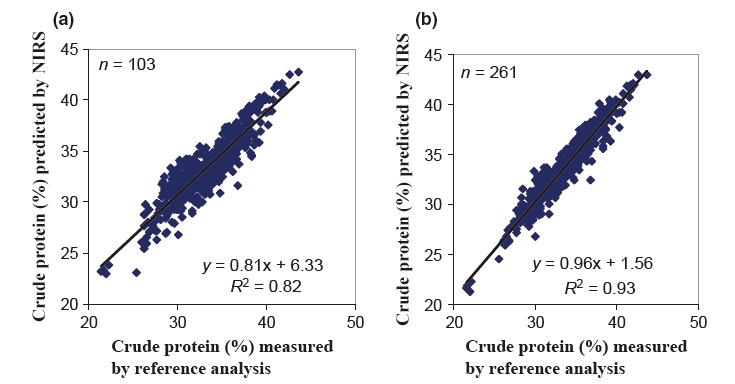
Scatter plot of crude protein (CP, in%) content in ground cowpea leaves collected from Tanzania, Kenya, Uganda, and Malawi, as well as under greenhouse and outdoor conditions in Göttingen, Germany, measured by means of reference (chemical) analysis and predicted by nondestructive near-infrared reflectance spectroscopy using a modified partial least-squares regression for (a) initial calibration model (Tefera 2006); (b) new calibration model developed in the present study.

The calibration statistics for CP (Tables 3 and 4) suggests that NIRS can predict this parameter in a wide range of cowpea leaves from different agro-ecological zones of East Africa with high accuracy (broad-based calibration). Okonya (2009) used the new calibration model to predict CP content in a set of cowpea leaf samples collected from Uganda (*n* = 42) and obtained reasonably good results of prediction (Table 4). The current findings are in agreement with those of other researchers (Míka et al. 2003; Stuth et al. 2003) that the use of sample sets with a wide range of chemical, environmental, and physical characteristics reduces the standard error and biases of the calibration models developed. Nevertheless, some sample sets could not be predicted satisfactorily, such as those from Malawi (Table 4) or the oven-dried set from Germany (Table 4) that all continued to have either very low *R*^2^ values or unacceptably high global-H values (>3). The dissimilarity of these samples for prediction is not surprising, when examining their location in the 3D space of the PCA (Fig. 2). In addition, the inclusion of some lablab samples in the sample set from Germany could have caused the problems with the prediction of the set using both the initial and new calibration models; however, the initial calibration (Tefera 2006) also included a few lablab samples (Table 4). The spectra from Dodoma samples, however, had been considered for the selection of the sample set for use in the new calibration model, and they appeared to be inconspicuous. Yet, their predicted CP contents are completely unsatisfactory (Table 4); however, no explanation for this has been found as yet. As samples for the development of calibrations were predominantly sun-dried, oven-drying might have changed the physical appearance of samples. Such potential production of outliers needs to be taken into consideration for future expansion of the calibration, as routine sample processing for laboratory analysis usually includes oven-drying. Figure 3 shows scatter plots of CP content measured using reference (chemical) analysis and NIRS predicted using a MPLS regression for both the initial and new model, respectively. The relationship between the reference values and the NIRS-predicted values only had a minor bias, with a slope close to 1 and a small intercept, clearly improved for the new calibration model. Stuth et al. (2003) stressed that NIRS predictive models are always a work in progress, and therefore, the model – although more robust – can probably be improved upon with more data.

## Conclusions

The NIR calibration results for CP in the different cowpea leaf sample sets showed a wide variation in CP composition as expected due to environmental differences, genetic materials, seasons, and growth stages of the plants in the samples used to develop the NIRS calibration models. NIRS analysis improved when calibration sets were developed from samples that were selected to represent the broad range of environmental conditions. We conclude from the present results that this technique is a good alternative to chemical analysis for the determination of CP contents in leaf samples from cowpea in the African context, as one of the main advantages of NIRS is the large number of compounds that can be measured at once in the same sample, thus substantially reducing the cost per analysis. The current model is applicable in predicting the CP content of young cowpea leaves for human nutrition from different agro-ecological zones and genetic materials, as cowpea leaves are one of the popular vegetables in the region. The new NIRS calibration model – although more robust – can highly likely be improved upon with more comprehensive data because such models are always a work in progress. The future direction should, therefore, aim at expanding variation to develop further robustness in the current calibration model developed for young cowpea leaves by including cowpea leaf samples from across the continent.

## Acknowledgments

Financial support for this study by the German Federal Ministry for Economic Cooperation and Development (BMZ) (part of GIZ Project No. 2002.7860.6001.00) is gratefully acknowledged. T. A. Tefera, M. S. Kabululu, J. Magesa, C. Malidadi, J. S. Okonya, and R. G. Rwegasira are appreciated for sharing the NIRS data generated from the cowpea leaf samples of their respective studies performed within the ProNIVA project.
